# Peripheral Neuroprotective and Immunomodulatory Effects of 5α-Reductase Inhibitors in Parkinson’s Disease Models

**DOI:** 10.3389/fphar.2022.898067

**Published:** 2022-07-22

**Authors:** Andrée-Anne Poirier, Mélissa Côté, Hend Jarras, Nadhir Litim, Jérôme Lamontagne-Proulx, Sara Al-Sweidi, Marc Morissette, Asmaa Lachhab, Martin Pelletier, Thérèse Di Paolo, Denis Soulet

**Affiliations:** ^1^ Centre de Recherche du CHU de Québec-Université Laval, Québec City, QC, Canada; ^2^ Faculté de Pharmacie, Université Laval, Québec City, QC, Canada; ^3^ Faculté de Médecine, Université Laval, Québec City, QC, Canada; ^4^ Institut sur la Nutrition et les Aliments Fonctionnels (INAF), Université Laval, Québec City, QC, Canada

**Keywords:** dutasteride, enteric nervous system, female hormones, finasteride, gut, inflammation, mitochondria, MPTP

## Abstract

Gastrointestinal disorders in Parkinson’s disease (PD) have been associated with neuronal alteration in the plexus of the gut. We previously demonstrated the immunomodulatory effect of female hormones to treat enteric neurodegeneration in the 1-methyl-4-phenyl-1,2,3,6-tetrahydropyridine (MPTP) mouse model of PD. This study made the hypothesis of obtaining similar neuroprotection as with hormone treatments by affecting steroidogenesis with two 5α-reductase inhibitors, finasteride and dutasteride. These drugs are approved to treat benign prostatic hyperplasia and alopecia and display mitochondrial effects. In MPTP-treated mice, the dopaminergic and vasoactive intestinal peptide (VIP) neurons alteration was prevented by finasteride and dutasteride, while the increase in proinflammatory macrophages density was inhibited by dutasteride treatment but not finasteride. NF-κB response, oxidative stress, and nitric oxide and proinflammatory cytokines production *in vitro* were only prevented by dutasteride. In addition, mitochondrial production of free radicals, membrane depolarization, decreased basal respiration, and ATP production were inhibited by dutasteride, while finasteride had no effect. In conclusion, the present results indicate that dutasteride treatment prevents enteric neuronal damages in the MPTP mouse model, at least in part through anti-inflammatory and mitochondrial effects. This suggests that drug repurposing of dutasteride might be a promising avenue to treat enteric neuroinflammation in early PD.

## 1 Introduction

Several studies have demonstrated the bidirectional connection between brain and gut (Martin et al., 2018; Breen et al., 2019; Kowalski and Mulak, 2019). Hence, it is now clear that the intestine may play a major role in neurological diseases development such as Parkinson’s disease (PD) (Killinger et al., 2018; Nishiwaki et al., 2020; Klann et al., 2021). A decade before motor symptoms appear, the majority of PD patients experience an impairment of autonomic nervous function that may lead to gastrointestinal disorders such as constipation (Postuma et al., 2013; Poirier et al., 2016a). In the last decade, some studies have associated these problems with an early alteration of the enteric nervous system (ENS) during the neuropathogenesis (Fasano et al., 2015; Lee et al., 2018). Moreover, aggregates of alpha-synuclein (α-syn) were observed in multiple sections of the gastrointestinal tract up to 20 years before motor symptoms (Wakabayashi et al., 1990; Stokholm et al., 2016; Fenyi et al., 2019). In accordance with Braak’s hypothesis, studies have suggested that the disease could be initiated in the ENS by the pathological accumulation of α-syn aggregates and an increase of inflammation, and that both could spread to the central nervous system (CNS) (Braak et al., 2003; Hawkes et al., 2007; Challis et al., 2020).

Inflammation also plays a major role in the degeneration of dopamine (DA) neurons in the development of PD (Pajares et al., 2020). An increase in proinflammatory cytokines and activated microglia have been found in the brain of PD patients and their presence correlates with damage to DA neurons in the nigrostriatal pathway (Nagatsu et al., 2000; Badanjak et al., 2021). In parallel, proinflammatory cytokines have also been observed in the colon of PD patients and correlate with the progression of the disease (Devos et al., 2013; Perez-Pardo et al., 2019). In our previous publications, we have also shown the involvement of the innate immune system in the alteration of DA neurons in the myenteric plexus in the 1-methyl-4-phenyl-1,2,3,6-tetrahydropyridine (MPTP) mouse model (Côté et al., 2011; Côté et al., 2015b; Poirier et al., 2016b).

In addition, decades of research with animal models have led to the association between the pathology of PD and mitochondrial dysfunctions (Borsche et al., 2020; Malpartida et al., 2021). Studies exploring brain of PD patients’ postmortem have shown a significant increase of oxidative stress, possibly due to mitochondrial dysfunctions (Seet et al., 2010; Filomeni et al., 2015; Chang and Chen, 2020). Other reports have also revealed an accumulation of misfolded α-syn aggregates within mitochondria, inducing defects in cellular respiration and apoptosis of the cell (Wang et al., 2019; Park et al., 2020). For example, it is well known that MPTP toxic metabolite 1-methyl-4-phenylpyridinium (MPP^+^) inhibits complex I of the mitochondrial respiration chain, reducing ATP production and increasing the presence of oxidative stress (Gerlach et al., 1991; Subramaniam and Chesselet, 2013; Woo et al., 2021). These elements suggest that oxidative stress, which is particularly associated with mitochondria, plays a major role in the pathogenesis of PD.

Since PD affects more men than women (1.5:1 ratio), many studies have been performed to understand the sexual differences in this disease (Gillies et al., 2014; Picillo et al., 2017). It has been suggested that estrogen could be used as a neuroprotective agent since hormonal therapies have been associated with a decreased risk of PD (Song et al., 2020). In the MPTP mouse model, higher brain neurotoxicity was observed in male than female mice, suggesting that estrogen levels influence susceptibility to the toxin (Miller et al., 1998; Isenbrandt et al., 2021). Other studies have also shown a beneficial therapeutic effect of female sex steroids on CNS-damaged DA neurons in MPTP mice (Bourque et al., 2013; Bourque et al., 2014; Bourque et al., 2015; Bourque et al., 2016; Isenbrandt et al., 2021; Thadathil et al., 2021). In addition to its neuroprotective role, there is evidence that estrogen is also involved in the regulation of the immune system (Kovats, 2015).

It is well known that hormonal therapies induce various undesirable side effects (increased risk of breast cancer, venous thrombosis, and stroke) (Chen, 2009; Vinogradova et al., 2019). In order to increase the endogenous biosynthesis of estrogen and inhibit the metabolism of progesterone, we used finasteride and dutasteride, two 5α-reductase enzyme inhibitors already approved for the treatment of benign prostatic hyperplasia and alopecia (Zhou et al., 2019; Zhou et al., 2020). It has been proposed that inhibition of 5α-reductase would result in a higher conversion rate of testosterone into estradiol by the enzyme aromatase (Litim et al., 2015). A previous study showed that both 5α-reductase inhibitors finasteride and dutasteride prevent the decrease of testosterone levels in plasma caused by MPTP, but only dutasteride inhibited DAergic loss in the CNS (Litim et al., 2015). Of great interest, in MPTP-treated animals, Isenbrandt et al. have shown that the dutasteride-mediated neuroprotection observed in sham male mice was not seen in gonadectomized male mice. Thus, the 5α-reductase inhibition may require the presence of sex hormones to be neuroprotective (Isenbrandt et al., 2021). Interestingly, a report by Soskić et al. (2008) suggested an association between mitochondrial functions and neuroprotection against calcium overload by finasteride and dutasteride. It is therefore possible that one mechanism of action of 5α-reductase inhibitors could involve the modulation of mitochondrial pore transition permeability through its interaction with the adenine nucleotide translocator 1/2 (ANT-1/2). The present study investigated the neuroprotective and immunomodulatory effects of finasteride and dutasteride in the myenteric plexus of the MPTP mouse model and the potential mechanisms involved in mitochondrial functions *in vitro*.

## 2 Materials and Methods

### 2.1 Animals

Ten weeks old C57BL/6 male mice were purchased from Charles River Canada (Montreal, QC, Canada). Animals were housed in cages under standard laboratory conditions, had access to food and water, and were acclimatized to a controlled temperature environment maintained under a 12-h light/dark cycle. Mice were handled in accordance with the National Institute of Health Guide for the Care and Use of Laboratory Animals, using a protocol approved by the Laval University Animal Care Committee. All efforts were made to minimize animal suffering and to reduce the number of mice used.

### 2.2 Treatments Administration

Mice were divided into six groups comprising 10 mice per group. Mice were treated once daily (s.i.d.) with vehicle (0.9% saline with 1% Tween 80 intraperitoneal [i.p.]), finasteride (5 or 12.5 mg/kg i.p.; Tocris, Ellisville, MO, United States), or dutasteride (5 or 12.5 mg/kg i.p.; Toronto Research Chemicals, Toronto, ON, Canada) for 10 days. On Day 5, mice received four injections of MPTP (6.5 mg/kg i.p.; Sigma-Aldrich, St. Louis, MO) at 2-h intervals, whereas the control groups received a saline solution. On Day 11, mice were anesthetized with isoflurane and killed to collect the brain and gut. The effects of finasteride and dutasteride treatment were previously reported to be neuroprotective against MPTP in the brain of these mice (Litim et al., 2015).

### 2.3 Tissue Preparation

Guts collection and preparation method was previously described (Côté et al., 2011). Briefly, guts were removed and placed 48 h in phosphate-buffered saline (PSB) containing 4% paraformaldehyde (PFA), pH 7.4. For each animal, the ileum was excised and microdissected to isolate the myenteric plexus. More precisely, the submucosal layer and the circular muscle layer were separated from the longitudinal muscle layer on which the myenteric plexus is attached.

### 2.4 Immunohistochemistry

As previously reported, DA neurons and macrophages of the myenteric plexus were identified on free-floating sections following immunohistochemistry (Côté et al., 2011). Briefly, myenteric ganglia neurons were stained with Cuprolinic blue for 60 min at 37°C (Holst and Powley, 1995). DA neurons and macrophages were identified with a polyclonal tyrosine hydroxylase antibody (TH; 1:1000; Cedarlane, ON, Canada) and the pan-macrophage/microglia marker ionized calcium-binding adaptor molecule 1 (Iba-1; 1:1000; Cedarlane), respectively. Biotinylated goat anti-rabbit IgG (1:1000; Cedarlane) was used as a secondary antibody. The signal was revealed with the ABC Vectastain Elite kit (Vector Laboratories, Inc., ON, Canada) and 3,3′-diaminobenzidine (DAB, Vector Laboratories, Inc.) as chromagen. All pictures were acquired with a Nikon C80i microscope equipped with a MicroFire digital camera (MBF Bioscience, Williston, VT). Figures were assembled using Adobe Illustrator CS3.

### 2.5 Immunofluorescence

VIP intensity and proinflammatory macrophage density were measured using an immunofluorescence-based approach. Free-floating tissues were incubated overnight at room temperature with a monoclonal rat antibody for the polyclonal rabbit VIP antibody (1:200; ImmunoStar, WI, United States) or the major histocompatibility complex (MHC) class II molecule (MHCII; 1:500; BD Biosciences Pharmingen). Myenteric plexus were stained with a donkey anti-rat secondary antibody conjugated to Alexa Fluor 488 (1:1000; Invitrogen Corporation, CA, United States) the next day. Nuclei were counterstained with 0.022% DAPI (Invitrogen). Images were taken using a confocal laser-scanning microscope (Olympus IX81-FV1000; ON, Canada) with a two-frame Kalman filter and several focal plans were acquired with a ×20 objective lens to generate maximum intensity projections.

### 2.6 Image Analysis

Stereological analyses were performed with the Stereo Investigator software 6.0 (Microbrightfield, VT, United States) operated by an observer (blind to treatments), as previously described (Côté et al., 2011). The contours of the whole mounted sections were traced as a virtual overlay over images through a ×4 objective lens in brightfield mode (Nikon C80i microscope equipped with a MicroFire digital camera; MBF Bioscience, Williston, VT). TH^+^ and Iba-1^+^ cell bodies were counted only if they did not cross the forbidden contours. Densities were calculated as the number of cells divided by the traced area (in mm^2^). MHCII and VIP quantifications were performed using the NIH ImageJ software (Wayne Rasband, Bethesda, MD, United States). Section contours were also traced as a virtual overlay on images with the freehand selection option. VIP immunofluorescent staining quantification was executed on gray scale values images and measured by the mean pixel intensity. MHCII^+^ cell count was performed using the embedded cell counter plugin and densities were calculated as the number of cells per area unit (in mm^2^). All figures were assembled using Adobe Illustrator CS3.

### 2.7 Cell Culture and Stimulation Protocol

Three different immortal cell types were used in this study: human neuroblastoma SH-SY5Y cells (ATCC, Manassas, VA, United States), human monocytic THP-1 cells (ATCC), and THP1-XBlue cells (derived from THP-1; InvivoGen, CA, United States). These cell lines were grown at 37°C in a humidified incubator of 5% CO_2_ in RPMI 1640 (Sigma) supplemented with 10% (v/v) heat-inactivated fetal bovine serum (FBS; Sigma), 100 μg/ml Normocin (InvivoGen), 100 units/ml penicillin/streptomycin (Sigma), and 200 μg/ml of Zeocin (InvivoGen). Cell density was maintained <2 × 10^6^ cells/ml and used before passage 20.

Experiments with *in vitro* treatments were performed as described previously (Côté et al., 2015a; Poirier et al., 2016b). Cells were centrifuged for 5 min at 300*g* and washed with fresh medium. They were then plated at a density of 5 × 10^5^ per well in a 96-well plate and incubated for 48 h with 200 nM phorbol 12-myristate 13-acetate (PMA; Sigma) to induce their differentiation. Cell medium was changed before incorporating finasteride (1 µM) or dutasteride (1 µM) to the cells for 24 h at 37°C; the doses of finasteride and dutasteride were as used by Soskić et al. (2008) in their cell culture studies. The next day, 1-methyl-4-phenylpyridinium (MPP^+^; 500 µM; Sigma-Aldrich Chemical) was added to cell media for 18 h. Supernatants were used for nuclear factor-kappa B (NF-κB), IL-1β, IL-6, and nitrite assays, whereas cells were used for the MTT viability test, cellular oxidative stress, mitochondrial free radicals production, mitochondrial membrane potential measurements, and seahorse analysis.

### 2.8 MTT Viability Validation

Cell viability test was performed by adding 100 μl of a 12-mM sterile filtered 3-(4,5-dimethylthiazolyl)-2,5-diphenyl-tetrazolium bromide (MTT; Thermo Fisher Scientific, Waltham, MA, United States) solution to each well. Cells were further incubated for 4 h in 5% CO_2_ at 37°C, allowing them to form the blue formazan product. After the removal of 75 µl of cell supernatant, 50 μl of 100% DMSO was added and plates were then gently swirled for 10 min at room temperature to dissolve the precipitate. Optical density (OD_540_) was monitored using a spectrophotometer (Synergy HT Multi-Mode Microplate Reader, Biotek, VT, United States).

### 2.9 Nitric Oxide Measurement

Griess reagent (Promega, Madison, WI) was used to determine nitric oxide (NO) contractions in the supernatants. Following treatments and MPP^+^ incubation, THP-1 supernatants were added with equal volumes of sulfanilamide solution (1% sulfanilamide in 5% H_3_PO_4_) for 10 min, and then, 0.1% of *N*-(1-napthyl) ethylenediamine dihydrochloride in water for an additional 10 min. Subsequently, optical density (OD_535_) was measured using a spectrophotometer (Biotek).

### 2.10 Nuclear Factor-Kappa B Assay

THP1-XBlue cells (InvivoGen) are THP-1 cells stably transfected with an NF-κB/AP-1 inducible reporter construct expressing a secreted embryonic alkaline phosphatase (SEAP) for monitoring NF-κB and AP-1 activation. After treatments and incubation, NF-κB activity was determined by adding QUANTI-Blue (InvivoGen) as a substrate of SEAP in the supernatants. Further incubation was pursued for 8 h at 37°C. The absorbance was then measured at 633 nm by spectrophotometry.

### 2.11 Interleukin-1β and Interleukin-6 Quantification

Levels of IL-1β and IL-6 proinflammatory cytokines were detected with enzyme-linked immunosorbent assays (ELISAs). The cytokines concentrations of treated and untreated THP-1 supernatants were measured in triplicate using the Human Quantikine ELISA kit (R&D Systems, MN). IL-1β and IL-6 levels were measured using a spectrophotometer. Data at 540 nm were subtracted from the data at 450 nm, according to the manufacturer’s instructions.

### 2.12 Flow Cytometry Analysis

Cellular oxidative stress was quantified by flow cytometry with CellROX Orange reagent (Thermo Fisher Scientific), a membrane-permeable reactive oxygen species (ROS)-sensitive probe. After plating THP-1 or SH-SY5Y cells, they were incubated with PMA and treated with finasteride, dutasteride, MPP^+^ as described previously (Section 2.7). Then, 5 µM CellROX Orange was added to cell culture medium and stained for 30 min at 37°C in the dark. At least 1 × 10^6^ cells/treatment were mechanically removed from the plate and were collected by centrifugation (500*g* for 5 min). Cells were then washed twice and resuspended in ice-cold PBS for flow cytometry analysis (488 nm excitation wavelength with 585/42 nm bandpass filter).

Mitochondrial free radicals were measured using MitoSOX Red (Thermo Fisher Scientific), a fluorescent probe specific for mitochondrial superoxide. THP-1 cells were plated and incubated with PMA for 48 h as described in Section 2.7. Cell medium was changed before incorporating finasteride or dutasteride (1 µM) to the cells for 24 h at 37°C. The next day, a combination of MPP^+^ (500 µM) with 5 µM MitoSox Red diluted in Dulbecco’s phosphate-buffered saline (D-PBS) was added to the cell media for 6 h (37°C in the dark). This combination avoids the MitoSOX Red saturation due to continuous ROS accumulation in the mitochondria. The incubation time of 6 h was determined by a time course experiment. At least 1 × 10^6^ cells/treatment were mechanically removed from the plate and were collected by centrifugation (500*g* for 5 min). Cells were then washed twice and resuspended in ice-cold PBS for flow cytometry analysis (488 nm excitation wavelength with 585/42 nm bandpass filter).

Flow cytometry was also used to analyze mitochondrial membrane potential with the fluorescent probe JC-1 (Thermo Fisher Scientific). This lipophilic cationic dye accumulates in mitochondria in a membrane potential-dependent manner. In cells with polarized mitochondria membrane, JC-1 selectively enters the mitochondria and forms orange-red aggregates. These punctate orange-red fluorescent staining are replaced by diffuse green monomers fluorescence in the presence of depolarized mitochondrial membrane. The red/green (FL2/FL1) ratio of the two fluorescence intensities allows the comparative measurement of mitochondrial membrane potential. THP-1 cells were plated and incubated with PMA for 48 h as described in Section 2.7. Cell medium was changed before incorporating finasteride or dutasteride (1 µM) to the cells for 24 h at 37°C. The next day, 500 µM MPP^+^ was added to cell media for 6 h. This time point was determined by a time course experiment. Cells were incubated in the dark at 37°C with JC-1 dye (5 μg/ml) 30 min before the experiment, according to the manufacturer’s instructions. At least 1 × 10^6^ cells/treatment were mechanically removed from the plate and were collected by centrifugation (500*g* for 5 min). THP-1 cells were then washed twice and resuspended in ice-cold PBS for flow cytometry analysis (488 nm excitation wavelength with 530/30 nm and 585/42 nm bandpass filters).

All analyses were performed with BD FACSCantoII system (Becton Dickinson, San Jose, CA, United States) and the fluorescence properties of 10,000 cells were recorded for each sample. Data were analyzed using FlowJo version 8.7 (FlowJo LLC, Ashland, OR, United States). Results are expressed as number of positive cells or as mean fluorescence intensity (MFI) of 10,000 cells.

### 2.13 Mitochondrial Respiration

Real-time analyses of the oxygen consumption rate (OCR) were performed using an XFe-96 Extracellular Flux Analyzer (Agilent/Seahorse Bioscience). THP-1 or SH-SY5Y cells were seeded to confluence in XF cell plates (6 × 10^4^ THP-1 and 4.5 × 10^4^ SH-SY5Y cells/well) and incubated with PMA for 48 h as described in Section 2.7. Cell medium was changed before incorporating finasteride or dutasteride (1 µM) to the cells for 24 h at 37°C. The next day, MPP^+^ (125 µM on THP-1 and 50 µM on SH-SY5Y cells) was added to cell media for overnight incubation. Optimization of MPP^+^ doses was determined to identify the half maximal inhibitory concentration (IC_50_) and to choose the best concentration. Cells were washed and incubated in XF basal medium supplemented by 2 mM glutamine, 10 mM glucose, and 1 mM sodium pyruvate. Measurements of mitochondrial basal respiration and ATP production, following sequential injections of 1 µM oligomycin and 1 µM/0.1 µM rotenone/antimycin A, were performed as described previously (Pelletier et al., 2014). Four measures were recorded between injections. Mitochondrial basal respiration corresponds to the last rate measurement before first injection minus non-mitochondrial respiration rate. ATP production corresponds to the last rate measurement before oligomycin injection minus minimum rate measurement after oligomycin injection. All data were analyzed using Wave 2.3 software and the Seahorse XF Cell Mito Stress Test Report Generator.

### 2.14 Statistics

Statistical analyses of in vivo experiments were performed using a one-way analysis of variance (ANOVA) followed by a Bonferroni post-test (95% confidence intervals), while *in vitro* analyses were performed using a two-way ANOVA (MPP^+^ toxin and 5α-reductase inhibitors treatments as independent variables) followed by a Bonferroni post-test (95% confidence intervals). All treatment comparisons were performed with GraphPad Prism 7.0 (GraphPad Software Inc., CA, United States). Data are presented as group mean ± SEM in all histograms. Statistical significance was determined with a threshold value of *p* < 0.05.

## 3 Results

### 3.1 Finasteride and Dutasteride Treatments Prevented 1-Methyl-4-Phenyl-1,2,3,6-Tetrahydropyridine-Induced Myenteric Dopamine Neuron Damage

**FIGURE 1:**
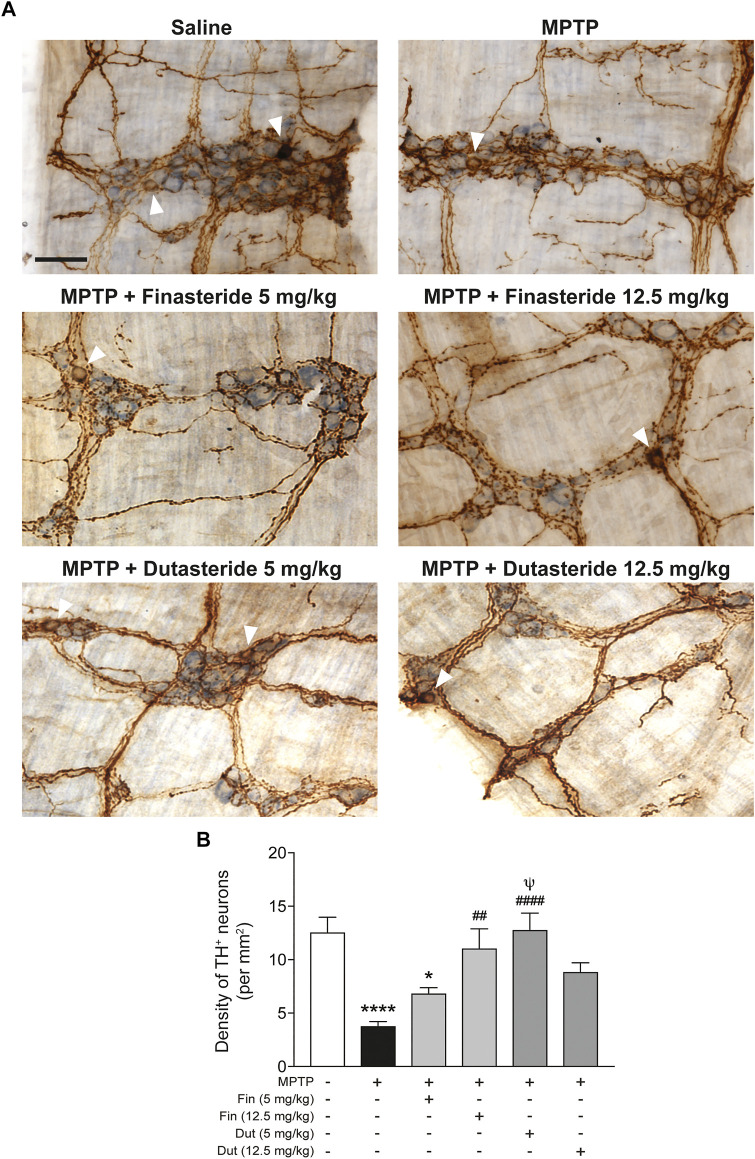
Finasteride and dutasteride treatments prevented MPTP-induced DA neurons damage in the myenteric plexus. **(A)** Photomicrographs of neuronal ganglia (light blue staining) and TH immunochemistry staining (brown DAB staining) in the myenteric plexus of the distal ileum. The white arrowheads indicate TH^+^ cell bodies; scale bar = 25 µm. **(B)** Stereological counts of TH^+^ neurons in the myenteric plexus showed neuroprotective effect of finasteride (Fin) (12.5 mg/kg) and dutasteride (Dut) (5 mg/kg) in MPTP mice. Values shown are the average densities (per mm^2^) ± SEM of 10 mice per group. ^*^ *p* < 0.05 and ^****^ *p* < 0.0001 compared to saline control mice; ^##^ *p* < 0.01 and ^####^ *p* < 0.0001 compared to MPTP lesioned mice; ^ψ^ *p* < 0.05 compared to MPTP + finasteride (5 mg/kg) treated mice.

Total neuronal ganglia density (light blue staining) and TH^+^ neurons density immunochemistry staining (brown DAB staining) were measured in the myenteric plexus of distal ileum of MPTP lesioned mice to seek their possible neuroprotection by 5α-reductase inhibitors. Figure 1A shows examples for each experimental group of mice and Figure 1B shows histograms of group means. The MPTP lesion and the finasteride and dutasteride treatments changed significantly the TH^+^ neuron density (*F*(5, 53) = 8.267, *p* < 0.0001) (Figure 1B). The MPTP lesion induced an extensive loss of TH^+^ neuron density. Finasteride treatment at the higher dose (investigated at 12.5 mg/kg) but not at the lower dose (5 mg/kg) prevented the MPTP-induced loss of TH^+^ neurons (Figure 1B). Dutasteride treatment at the lower dose (tested at 5 mg/kg) completely prevented the MPTP-induced loss of TH^+^ neurons, whereas at the higher dose (tested at 12.5 mg/kg), the TH^+^ neuron density was neither different from controls nor vehicle-treated MPTP-lesioned mice (Figure 1B).

### 3.2 Finasteride and Dutasteride Treatments Prevented 1-Methyl-4-Phenyl-1,2,3,6-Tetrahydropyridine-Induced Vasoactive Intestinal Peptide Neuronal Loss in the Myenteric Plexus

**FIGURE 2:**
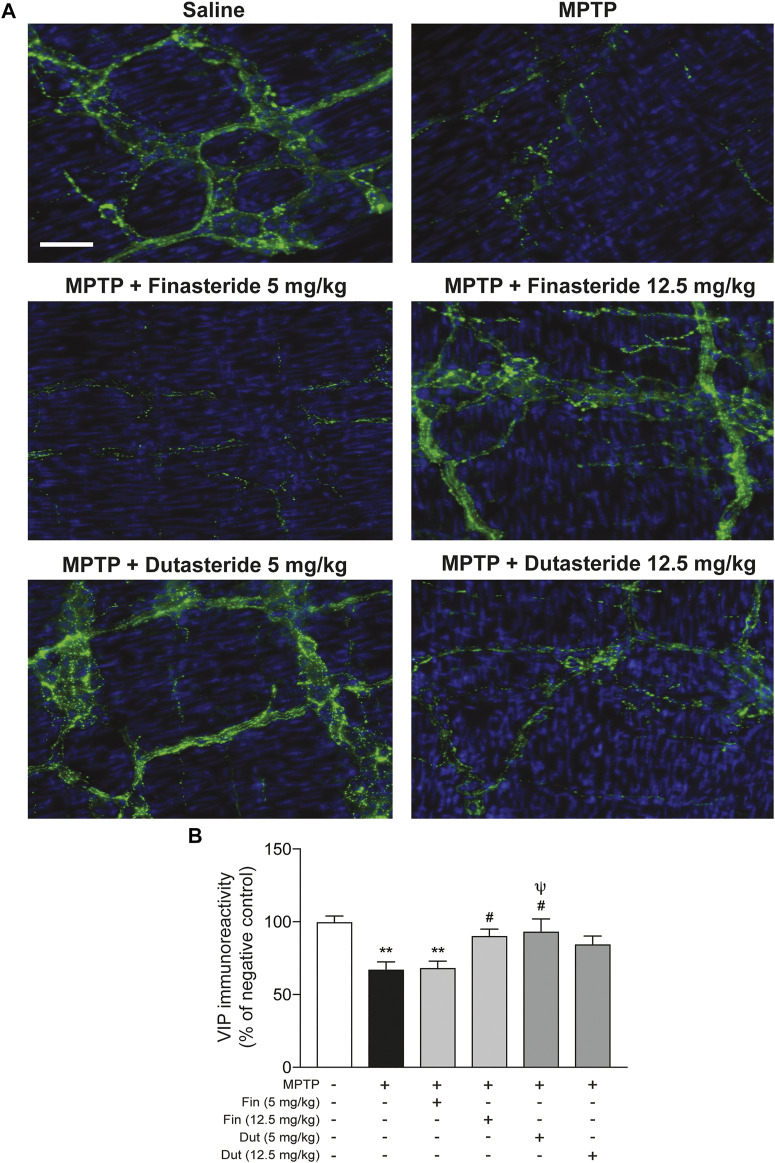
Protection of VIP neurons in the myenteric plexus of MPTP mice with finasteride and dutasteride. **(A)** Photomicrographs of VIP immunofluorescence staining in the myenteric plexus of the distal ileum; scale bar = 25 µm. **(B)** VIP immunoreactivity showed neuroprotective effect of finasteride (Fin) (12.5 mg/kg) and dutasteride (Dut) (5 mg/kg) in MPTP mice. Values shown are the average densities (per mm^2^) ± SEM of 8–10 mice per group. ^**^ *p* < 0.01 compared to saline controls mice; ^#^ *p* < 0.05 compared to MPTP lesioned mice; ^ψ^ *p* < 0.05 compared to MPTP + finasteride (5 mg/kg) treated mice.

Our previous studies have shown changes of the neuropeptide VIP in the mouse myenteric plexus associated with estrogen neuroprotection against MPTP toxicity (Côté et al., 2015a; Côté et al., 2015b). Hence, VIP neurons were next investigated in MPTP mice that received 5α-reductase inhibitor treatment. Figure 2A shows photomicrographs of VIP immunofluorescence staining in the myenteric plexus of the distal ileum of each experimental group of mice. The MPTP lesion and the finasteride and dutasteride treatments affected VIP immunoreactivity (*F*(5, 48) = 6.607; *p* < 0.0001) (Figure 2B). As for TH^+^ neuron density, VIP immunoreactivity was decreased in the vehicle-treated MPTP mice (Figure 2B). Also, as for TH^+^ neuron density, the VIP immunoreactivity decrease by MPTP was prevented by finasteride treatment at 12.5 mg/kg (but not at 5 mg/kg) and dutasteride treatment at 5 mg/kg in MPTP mice (Figure 2B). Moreover, as for TH^+^ neuron density, dutasteride treatment at 12.5 mg/kg was neither different from controls nor vehicle-treated MPTP mice (Figure 2B).

### 3.3 Dutasteride Treatment Prevented the 1-Methyl-4-Phenyl-1,2,3,6-Tetrahydropyridine-Induced Elevation of Macrophage Density in the Myenteric Plexus

**FIGURE 3:**
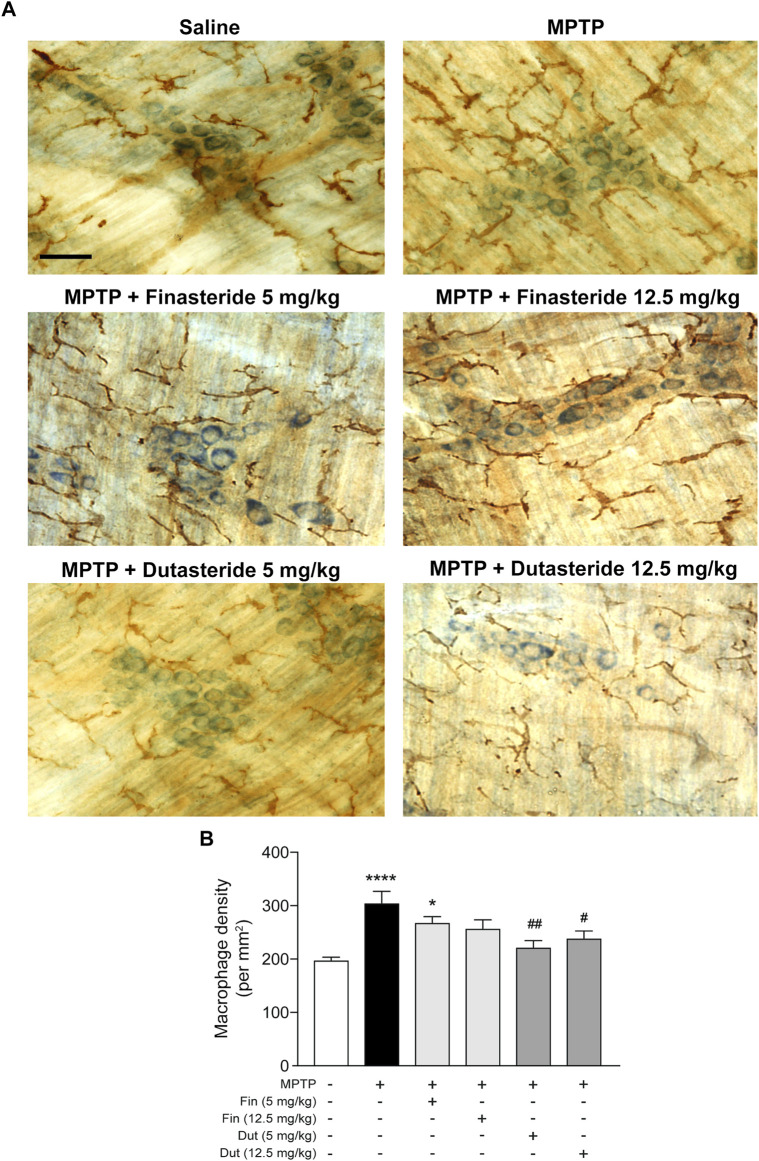
Dutasteride treatment prevented the MPTP-induced macrophage response in the myenteric plexus, whereas finasteride gave an inconclusive effect. **(A)** Photomicrographs of Iba-1 immunochemistry staining (brown DAB staining) in the myenteric plexus of the distal ileum; scale bar = 25 µm. **(B)** Stereological counts of macrophages in the myenteric plexus showed an MPTP-induced increase in macrophage density inhibited by dutasteride (Dut) treatment (5 and 12.5 mg/kg), whereas finasteride (Fin) treatment at 5 mg/kg was ineffective and at 12.5 mg/kg led to no change compared to controls or vehicle-treated MPTP mice. Values shown are the average densities (per mm^2^) ± SEM of 10 mice per group. ^*^ *p* < 0.05 and ^****^ *p* < 0.0001 compared to saline control mice; ^#^ *p* < 0.05 and ^##^ *p* < 0.01 compared to MPTP lesioned mice.

MPTP-lesioned mice exhibit an activation of the innate immune response due to infiltration of macrophages in the myenteric plexus (Côté et al., 2015b). We previously showed that estrogenic drugs inhibit this activation (Côté et al., 2015a; Poirier et al., 2016b). The impact of 5α-reductase inhibitors on this immune response is unknown and was next investigated using the pan-macrophage/microglia marker Iba-1 immunochemistry staining (brown DAB staining) in the myenteric plexus of distal ileum. Figure 3A shows examples of photomicrographs of each experimental group of mice. The MPTP lesion and the finasteride and dutasteride treatments affected macrophage density (*F*(5, 54) = 7.065; *p* < 0.0001) (Figure 3B). The MPTP lesion led to an increase of macrophage density that remained elevated in MPTP mice treated with finasteride at 5 mg/kg, while a concentration of 12.5 mg/kg reduced partially the density (Figure 3B). Dutasteride treatment at both doses significantly inhibited the lesion-induced increase of macrophage density (Figure 3B).

### 3.4 Proinflammatory Myenteric Macrophages Density Increased by 1-Methyl-4-Phenyl-1,2,3,6-Tetrahydropyridine Was Reduced by Dutasteride Treatment

**FIGURE 4:**
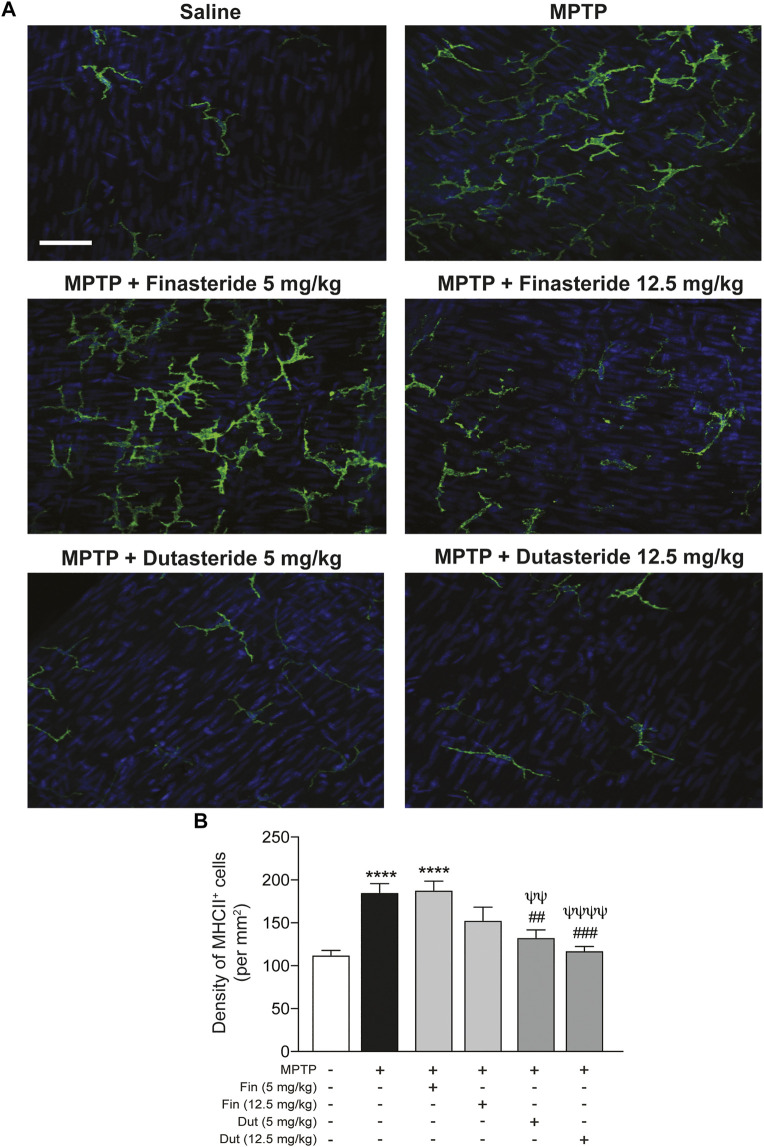
Proinflammatory macrophages density increased by MPTP was reduced by dutasteride treatment in the myenteric plexus, whereas finasteride gave an inconclusive effect. **(A)** Photomicrographs of MHCII immunofluorescence labeling in the myenteric plexus of the distal ileum; scale bar = 25 µm. **(B)** Stereological counts of MHCII^+^ cells showed that dutasteride (Dut) (5 and 12.5 mg/kg) prevented the increase of proinflammatory macrophages in the myenteric plexus of MPTP mice, whereas finasteride treatment at 5 mg/kg was ineffective and at 12.5 mg/kg led to no change compared to controls or vehicle-treated MPTP mice. Values shown are the average densities (per mm^2^) ± SEM of 10 mice per group. ^****^ *p* < 0.0001 compared to saline control mice; ^##^ *p* < 0.01 and ^###^ *p* < 0.001 compared to MPTP lesioned mice; ^ψψ^ *p* < 0.01 and ^ψψψψ^ *p* < 0.0001 compared to MPTP + finasteride (5 mg/kg) treated mice.

Macrophages differentially express cell surface receptors allowing distinguishing their phenotypes; proinflammatory macrophages express high levels of MHC class II receptors (Stöger et al., 2012). We then explored the effect of 5α-reductase inhibitors on the expression of major histocompatibility complex (MHC) class II (abbreviated to MHCII) receptors in the myenteric plexus of the MPTP-treated mice. Figure 4A shows examples of photomicrographs of each experimental group of mice. The MPTP lesion and the finasteride and dutasteride treatments affected MHCII receptors density (*F*(5, 54) = 11.09; *p* < 0.0001) (Figure 4B). Similarly to the results for Iba-1, the MPTP lesion led to an increase of MHCII receptors density that remained elevated in MPTP mice treated with finasteride 5 mg/kg, while at 12.5 mg/kg this density was not significantly different from the density of control or vehicle-treated MPTP mice (Figure 4B). Dutasteride treatment at both doses significantly inhibited the MPTP-induced increase of MHCII receptors density (Figure 4B).

### 3.5 Dutasteride Treatment Inhibited the Induction of Nuclear Factor-Kappa B Proinflammatory Response by 1-Methyl-4-Phenylpyridinium in a Human Monocytic Cell Line

**FIGURE 5:**
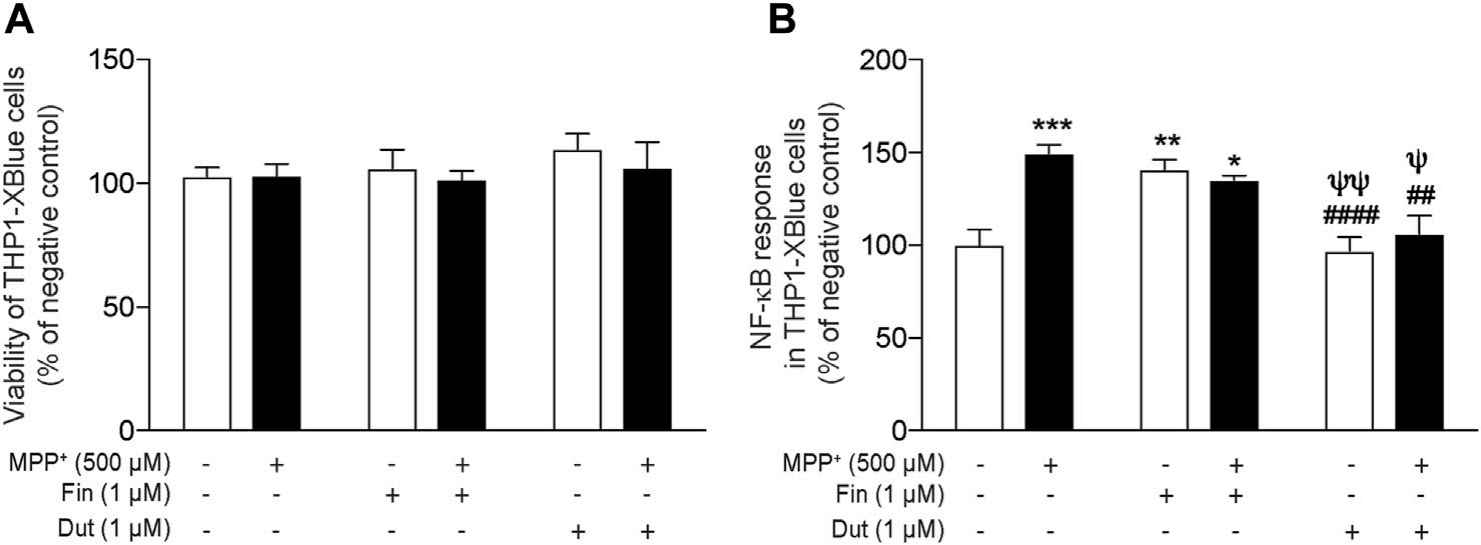
Dutasteride but not finasteride treatment inhibited the induction of NF-κB proinflammatory response by MPP^+^ in a human monocytic cell line. NF-κB activity was determined in the supernatant of monocytic cells treated with finasteride (Fin) or dutasteride (Dut) combined or not with the MPP^+^ toxin. Using THP1-XBlue cells containing NF-κB reporter gene allowed the evaluation of NF-κB activation level in this human cell line. Histogram show **(A)** THP1-XBlue cell viability quantification (MTT assay) and **(B)** NF-κB response as percentage of negative control. Results are the mean of three experiments. ^*^ *p* < 0.05, ^**^ *p* < 0.01, ^***^ *p* < 0.001 compared to control; ^##^ *p* < 0.01 and ^####^ *p* < 0.0001 compared to MPP^+^ treatment; ^ψ^ *p* < 0.05 and ^ψψ^ *p* < 0.01 compared to finasteride only.

The anti-inflammatory activity of the 5α-reductase inhibitors was next investigated *in vitro* with the THP1-XBlue cell line with a NF-κB reporter gene. We previously showed that the MPTP metabolite, MPP^+^, induces an inflammatory response that upregulates the NF-κB response (Côté et al., 2015a, Côté et al., 2015b; Poirier et al., 2016b). NF-κB activity was determined in the supernatant of monocytic cells treated with finasteride or dutasteride with or without the MPP^+^ toxin (Figure 5). THP1-XBlue cell viability quantified using the MTT assay showed no effect of the MPP^+^ toxin and/or the finasteride or dutasteride treatments (effect of MPP^+^: *F*(1, 30) = 0.5591, *p* = 0.4604; effect of the 5α-reductase inhibitors treatments: *F*(2, 30) = 0.7128, *p* = 0.4984; their interaction: *F*(2, 30) = 0.1846, *p* = 0.8324) (Figure 5A). However, the MPP^+^ and finasteride or dutasteride treatments affected the NF-κB response (effect of MPP^+^: *F*(1, 30) = 10.46, *p* = 0.0030; effect of the 5α-reductase inhibitors treatments: *F*(2, 30) = 15.01, *p* < 0.0001; interaction: *F*(2, 30) = 8.912, *p* = 0.0009) (Figure 5B). The NF-κB response was increased by MPP^+^ (Figure 5B). Finasteride treatment alone also increased the NF-κB response and it remained elevated with MPP^+^ (Figure 5B). By contrast, dutasteride treatment in both control and MPP^+^ mice left the NF-κB response at a level similar to the control mice (Figure 5B).

### 3.6 Significant Reduction of Proinflammatory Markers by Dutasteride Treatment in 1-Methyl-4-Phenylpyridinium-Stimulated THP-1 Cells

**FIGURE 6:**
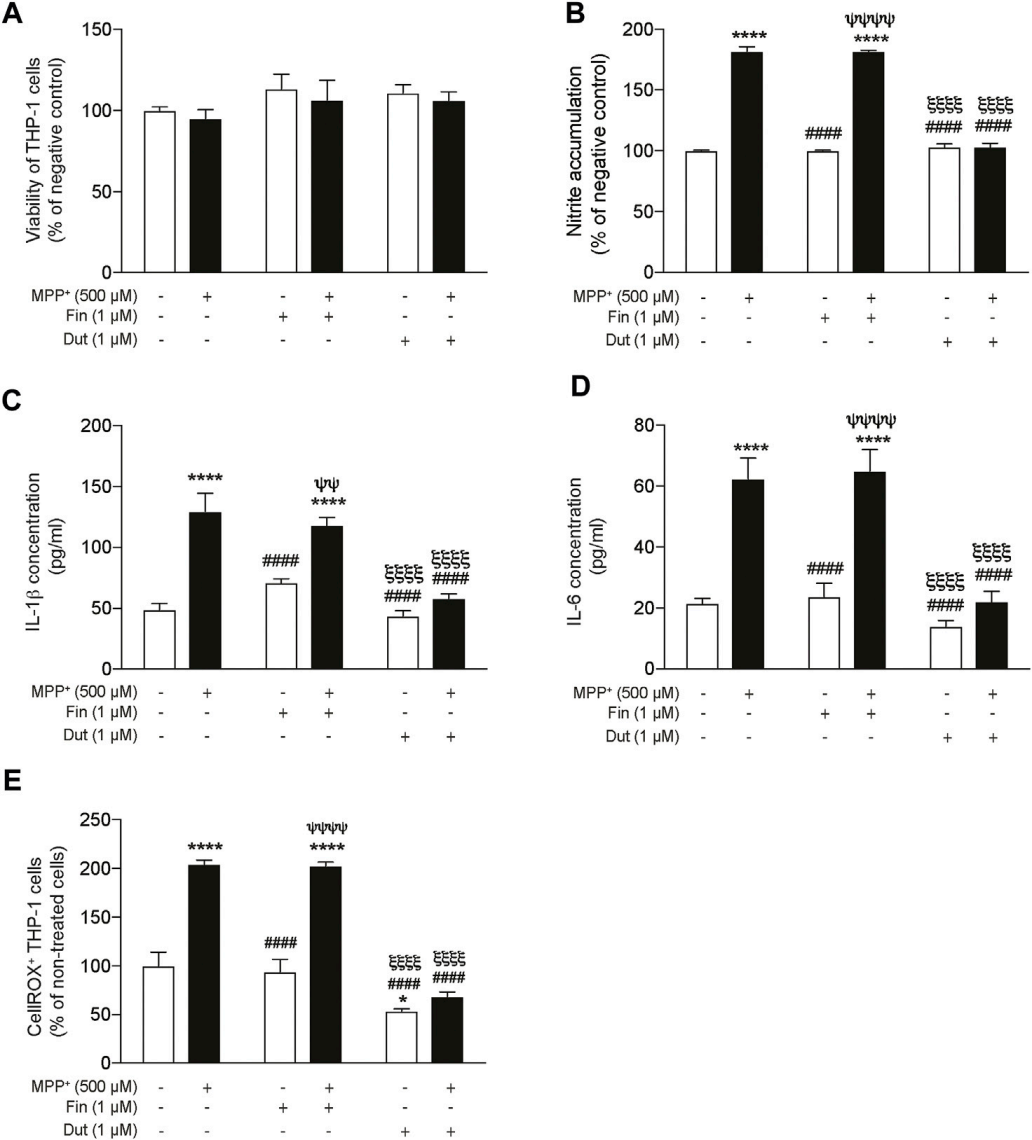
Significant reduction of proinflammatory markers by dutasteride but not by finasteride treatment in THP-1 cells following MPP^+^ stimulation. Nitric oxide (NO), IL-1β, and IL-6 production was measured in the supernatant of THP-1 monocytic cells treated with finasteride (Fin) or dutasteride (Dut) in the presence or the absence of MPP^+^. Histograms show **(A)** THP-1 cell viability quantification (MTT assay) and **(B)** NO accumulation by THP-1 cells (Griess assay) as percentage of negative control, **(C)** IL-1β and **(D)** IL-6 cytokine production in pg/ml, and **(E)** number of positive THP-1 cells to the oxidative stress marker CellROX Orange as percentage of cells. Results are the mean of three experiments. ^*^ *p* < 0.05 and ^****^ *p* < 0.0001 compared to control; ^####^ *p* < 0.0001 compared to MPP^+^ treatment; ^ψψ^ *p* < 0.01 and ^ψψψψ^ *p* < 0.0001 compared to Finasteride only; ^ξξξξ^ *p* < 0.0001 compared to MPP^+^ + finasteride.

The proinflammatory markers nitric oxide (NO), interleukin-1β (IL-1β), and interleukin-6 (IL-6) productions were measured in the supernatant of THP-1 monocytic cells treated with finasteride or dutasteride in the presence or the absence of MPP^+^ (Figure 6). THP-1 monocytic cell viability quantified using the MTT assay showed no effect of MPP^+^ and/or the finasteride or dutasteride treatments or their interaction (effect of MPP^+^: *F*(1, 12) = 0.8832, *p* = 0.3658; effect of 5α-reductase inhibitors: *F*(2, 12) = 1.759, *p* = 0.2139; their interaction: *F*(2, 12) = 0.01573, *p* = 0.9844) (Figure 6A). However, we showed significant differences in THP-1 cells for NO accumulation (Griess assay) (effect of MPP^+^: *F*(1, 12) = 781.1, *p* < 0.0001; effect of 5α-reductase inhibitors: *F*(2, 12) = 168.7, *p* < 0.0001; their interaction: *F*(2, 12) = 195.3, *p* < 0.0001) (Figure 6B), IL-1β concentration (effect of MPP^+^: *F*(1, 30) = 58.74, *p* < 0.0001; effect of 5α-reductase inhibitors: *F*(2, 30) = 19.55, *p* < 0.0001; their interaction: *F*(2, 30) = 9.506, *p* = 0.0006) (Figure 6C), IL-6 concentration (effect of MPP^+^: *F*(1, 30) = 60.55, *p* < 0.0001; effect of 5α-reductase inhibitors: *F*(2, 30) = 18.89, *p* < 0.0001; their interaction: *F*(2, 30) = 8.109, *p* = 0.0015) (Figure 6D), and the oxidative stress marker CellROX (effect of MPP^+^: *F*(1, 12) = 119.8, *p* < 0.0001; effect of 5α-reductase inhibitors: *F*(2, 12) = 73.44, *p* < 0.0001; their interaction: *F*(2, 12) = 19.38, *p* = 0.0002) (Figure 6E). NO accumulation (Figure 6B), IL-1β concentration (Figure 6C), IL-6 concentration (Figure 6D), and the number of CellROX^+^ (Figure 6E) were all increased by MPP^+^, while both finasteride and dutasteride alone did not show difference. Interestingly, dutasteride treatment alone decreased the number of CellROX^+^ cells compared to controls. Finasteride treatment did not prevent the MPTP-induced increases of these makers, whereas dutasteride completely prevented them.

### 3.7 Impaired Mitochondrial Function Was Prevented by Dutasteride Treatment in 1-Methyl-4-Phenylpyridinium-Stimulated THP-1 Cells

**FIGURE 7:**
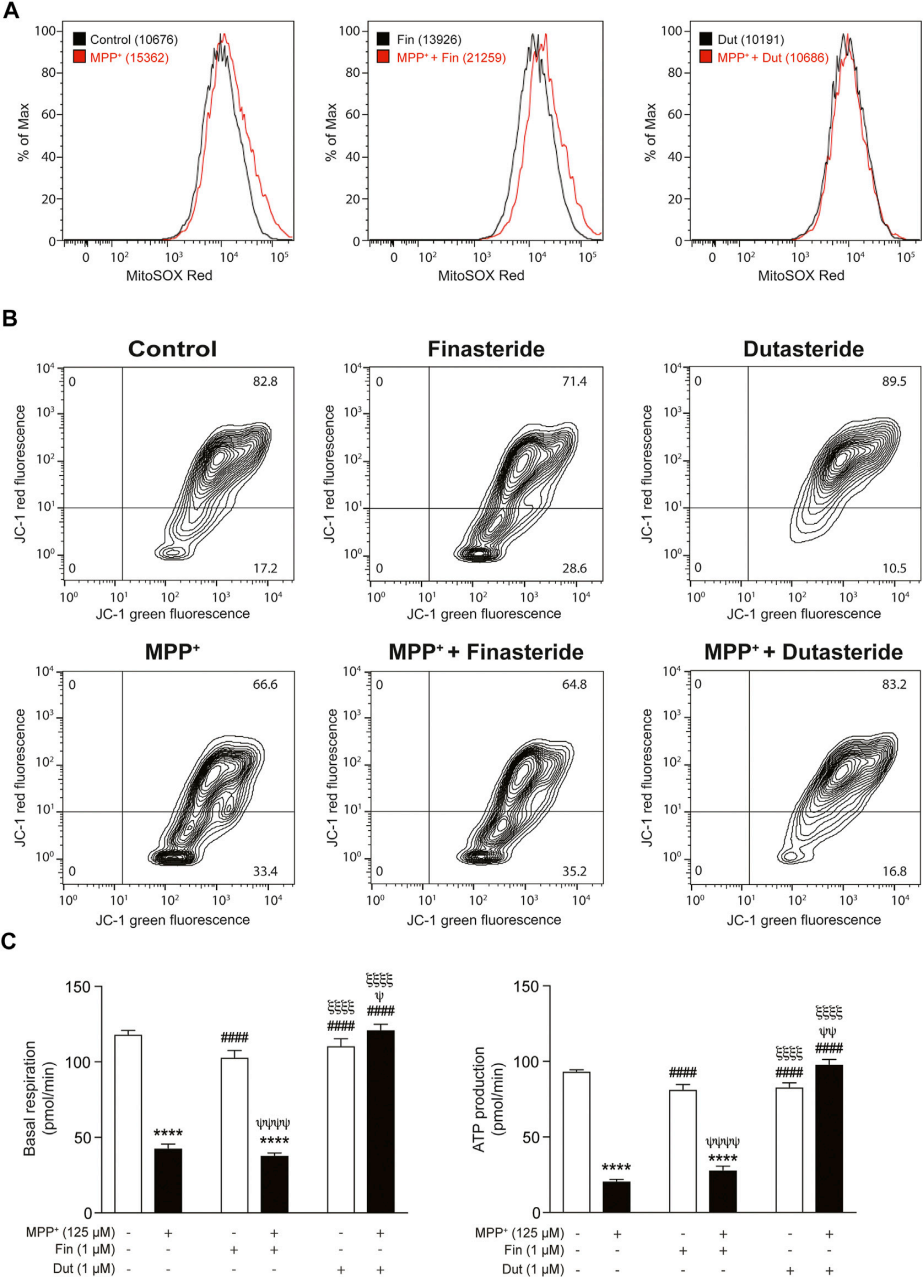
Impaired mitochondrial function was prevented by dutasteride but not by finasteride treatment in MPP^+^-stimulated THP-1 cells. Three parameters of mitochondrial function were evaluated with finasteride- (Fin) or dutasteride-treated (Dut) THP-1 cells exposed or not to the MPP^+^ toxin. Flow cytometry analyses were used to measure mitochondrial oxidative stress and membrane potential, while oxygen consumption rate (OCR) was quantified by Seahorse assays. **(A)** An increase in the mean fluorescence intensity (MFI; values in parentheses) of the marker MitoSOX red reagent corresponds to an increase in mitochondrial oxidative stress production (shift of the curve to the right). **(B)** The JC-1 dye enables evaluation of the mitochondrial membrane potential; JC-1 green intensity increases with membrane depolarization, while JC-1 red intensity increases with polarized membranes. **(C)** Histograms show mitochondrial basal respiration and ATP production quantification in each condition (pmol/min). Results are the mean of three experiments. ^****^ *p* < 0.0001 compared to control; ^####^ *p* < 0.0001 compared to MPP^+^ treatment; ^ψ^ *p* < 0.05, ^ψψ^ *p* < 0.01 and ^ψψψψ^ *p* < 0.0001 compared to finasteride only; ^ξξξξ^ *p* < 0.0001 compared to MPP^+^ + finasteride.

Three parameters of mitochondrial function were evaluated in THP-1 cells exposed or not to MPP^+^ with and without the 5α-reductase inhibitors treatments. Flow cytometry analyses were used to measure mitochondrial oxidative stress and membrane potential, while the oxygen consumption rate (OCR) was quantified by Seahorse assays. Dose–response of MPP^+^ in THP-1 and SH-SY5Y cells are shown in Supplementary Figure S1. Figure 7A shows the peak of fluorescence intensity of the marker MitoSOX Red. The left panel shows that peak intensity was shifted in the presence of MPP^+^, the middle panel reported fluorescence intensity in the presence of finasteride showing a change compared to MPP^+^, and the right panel shows that dutasteride prevented the MPP^+^-induced fluorescence shift. The MPP^+^ effect on this fluorescence corresponds to an increase in mitochondrial oxidative stress production that was opposed by dutasteride but not by finasteride. Figure 7B shows results of the JC-1 marker enabling evaluation of the mitochondrial membrane potential; fluorescence isothiocyanate (FITC) expression is trigged by a membrane depolarization, while PE expression represents polarized membranes. Finasteride, like MPP^+^ and finasteride + MPP^+^ treatments, led to a different pattern of depolarized membranes compared to the controls. By contrast, control, dutasteride, and dutasteride + MPP^+^ treatments showed similar patterns indicating that dutasteride opposed the effect of MPP^+^ and kept membranes more polarized. Histograms in Figure 7C show mitochondrial basal respiration and ATP production quantification that were significantly affected by MPP^+^ and finasteride and dutasteride treatments (basal respiration: effect of MPP^+^: *F*(1, 14) = 236.2, *p* < 0.0001; effect of 5α-reductase inhibitors: *F*(2, 14) = 95.78, *p* < 0.0001; their interaction: *F*(2, 14) = 92.64, *p* < 0.0001; ATP production: effect of MPP^+^: *F*(1, 14) = 289.4 *p* < 0.0001; effect of 5α-reductase inhibitors: *F*(2, 14) = 113.7, *p* < 0.0001; their interaction: *F*(2, 14) = 151.7 *p* < 0.0001). MPP^+^ significantly reduced basal respiration and ATP production of THP-1 cells. Finasteride or dutasteride treatments alone left these parameters at control values. Finasteride did not oppose the effect of MPP^+^, whereas dutasteride completely prevented the MPP^+^-induced reduction of basal respiration and ATP production.

SH-SY5Y neuroblastoma cells were also significantly affected by MPP^+^ and the finasteride or dutasteride treatments (Supplementary Figure S2A: effect of MPP^+^: *F*(1, 12) = 351.2, *p* < 0.0001; effect of 5α-reductase inhibitors: *F*(2, 12) = 18.57, *p* < 0.0002; their interaction: *F*(2, 12) = 1.694 *p* = 0.2249; Supplementary Figure S2B: basal respiration, effect of MPP^+^: *F*(1, 8) = 170.5 *p* < 0.0001; effect of 5α-reductase inhibitors: *F*(2, 8) = 32.88, *p* < 0.0001; their interaction: *F*(2, 8) = 3.684 *p* = 0.0734; and Supplementary Figure S2C: ATP production, effect of MPP^+^: *F*(1, 8) = 192.4 *p* < 0.0001; effect of 5α-reductase inhibitors: *F*(2, 8) = 34.53 *p* = 0.0001; their interaction: *F*(2, 8) = 6.389 *p* = 0.0220). The percentage of positive SH-SY5Y cells to the oxidative stress marker CellROX were increased in the presence of MPP^+^ (Supplementary Figure S2A). Mitochondrial basal respiration of the SH-SY5Y cells was reduced in the presence of MPP^+^, this was not prevented by the finasteride treatment, whereas MPP^+^- and dutasteride-treated cells showed no change compared to controls or MPP^+^ treatment alone (Supplementary Figure S2B). Interestingly, dutasteride treatment of the SH-SY5Y cell alone increased their basal respiration (Supplementary Figure S2B). ATP production in SH-SY cells was also reduced with MPP^+^ treatment alone and was not prevented in the finasteride and dutasteride treatments (Supplementary Figure S2C). Interestingly, dutasteride treatment of the SH-SY5Y cell alone increased their ATP production (Supplementary Figure S2C).

## 4 Discussion

**FIGURE 8:**
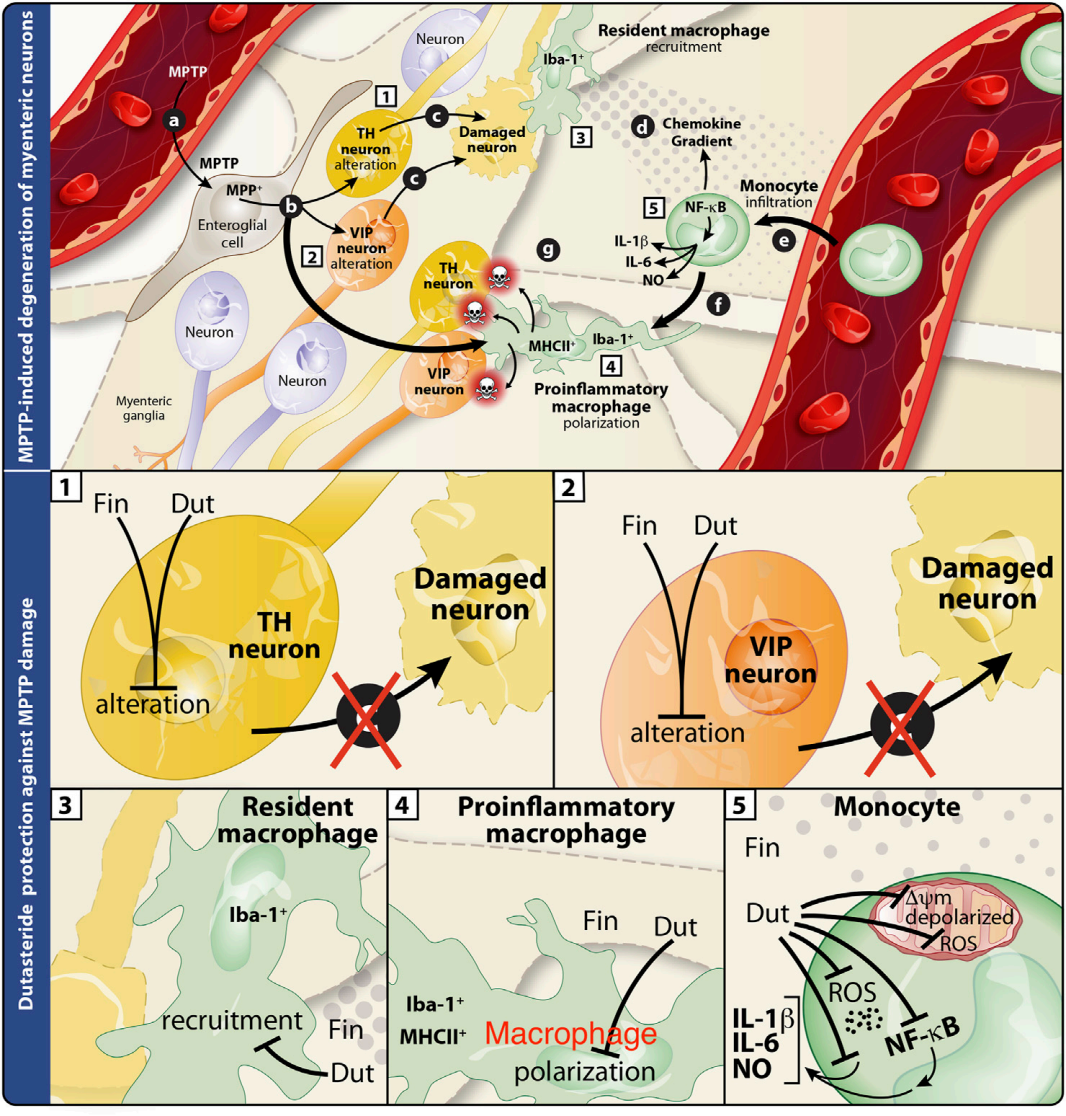
Summary of the effects of finasteride and dutasteride in the enteric nervous system of the MPTP mouse model of Parkinson’s disease. Schematic interpretation of the effect of finasteride (Fin) or dutasteride (Dut) treatments in the myenteric plexus of the MPTP mouse model according to the present results (illustration adapted from Côté et al., 2011). Upper panel illustrates the MPTP-induced damages in the myenteric plexus (previously investigated in (25)). **(A)** MPTP enters myenteric ganglia and is processed by enteroglial cells into MPP^+^, **(B)** the latter is recaptured by macrophages and leads to Iba-1^+^MHCII^+^ proinflammatory cell polarization, or by TH^+^ and VIP^+^ neurons and leads to cell damage **(C)**. This neuronal damage induces an immune response from Iba-1^+^ resident macrophages and the release of chemokines **(D)**. The resulting chemokine gradient promotes the detrimental infiltration of monocytes **(E)**. These events are associated with monocyte differentiation **(F)** into proinflammatory macrophages (Iba-1^+^MHCII^+^) that may subsequently induce neuronal damage **(G)**. Lower panels are close-ups of the individual effects of finasteride or dutasteride in each cell type studied. **(1)** MPTP-induced TH^+^ neuron alteration is completely inhibited by finasteride and dutasteride treatments. **(2)** Finasteride and dutasteride inhibit VIP^+^ neuron damage. Dutasteride administration inhibits both Iba-1^+^ resident macrophage recruitment **(3)** and Iba-1^+^MHCII^+^ proinflammatory macrophage polarization **(4)** following MPTP injections, while finasteride had no effect. **(5)** Finally, dutasteride treatment completely inhibits activation of the NF-κB transcription factor, cellular production of oxidative stress (ROS), production of the proinflammatory cytokines IL-1β and IL-6, mitochondrial membrane depolarization, and mitochondrial free radical production (ROS) in monocytes. The arrows indicate the consequences of administering MPTP, while the closed ended lines show an inhibition of the effect.

The present results showed a decrease of MPTP-induced lesion in DA and VIP neurons in the murine myenteric plexus. We show for the first time that treatment with finasteride (at the higher dose tested) and dutasteride (at the lower dose tested) can prevent neuronal damage in the gut. Total and proinflammatory enteric macrophages density increased by MPTP were prevented by the dutasteride treatment but not by finasteride. The effects of these 5α-reductase inhibitors were then investigated in two immortalized human cell lines. In the human monocytic cell line (THP1-XBlue cells containing NF-κB reporter gene), the MPP^+^ induced a proinflammatory response, as measured with an increase of NF-κB levels, was inhibited by dutasteride but not finasteride treatment. Furthermore, we observed an increase of the proinflammatory markers NO, IL-1β, and IL-6 mediated by MPP^+^ in THP1-XBlue cells. The increase of these markers was significantly prevented by the dutasteride but not the finasteride treatment. While the number of THP1-XBlue cells positive to the oxidative stress marker CellROX was increased by MPP^+^, the dutasteride treatment prevented the increase of these toxic markers. Finally, we showed that dutasteride, but not finasteride treatment, protected the THP1-XBlue cells from an alteration of free radicals production, membrane depolarization, basal respiration, and ATP production caused by the MPP^+^ treatment. For a summary of the results presented in this study, see Table 1 and Figure 8.

**TABLE 1:** Summary of the effects of finasteride and dutasteride treatments reported in this study.

Measure	Finasteride	Dutasteride
In vivo MP of MPTP mice		
*DA neurons*	Active at 12.5 mg/kg	Active at 5 mg/kg
Neuroprotection against MPTP		
*VIP neurons*	Active at 12.5 mg/kg	Active at 5 mg/kg
Neuroprotection against MPTP		
*Macrophage density*	Inconclusive	Active at 5 and 12.5 mg/kg
Neuroprotection against MPTP		
*Proinflammatory macrophage density*	Inconclusive	Active at 5 and 12.5 mg/kg
Neuroprotection against MPTP		
In vivo		
*Monocytic cells*		
Protection against MPP^+^		
NF-κB proinflammatory response	Inactive	Active
*THP-1 cells*		
Protection against MPP^+^		
Proinflammatory markers		
NO	Inactive	Active
IL-1β	Inactive	Active
IL-6	Inactive	Active
Oxidative stress marker CellROX	Inactive	Active
*THP-1 cells*		
Protection against MPP^+^		
Mitochondrial function		
Oxidative stress production	Inactive	Active
Mitochondrial membrane potential	Inactive	Active
Mitochondrial basal respiration	Inactive	Active
ATP production	Inactive	Active
*SH-SY5Y cells*		
Protection against MPP^+^		
Mitochondrial function		
Oxidative stress marker CellROX	Inactive	Inactive
Mitochondrial basal respiration	Inactive	Inconclusive
ATP production	Inactive	Inactive

Three types of 5α-reductase named 5α-reductase types 1, 2, and 3, display a different distribution in the body (Azzouni et al., 2012; Xiao et al., 2020). In the human brain, 5α-reductase subtype 3 mRNA expression level was reported to be the highest compared to subtypes 1 and 2 (Yamana et al., 2010). In the small intestine, show also a highest expression level of subtype 3, whereas expression levels of subtypes 1 and 2 were low (Yamana et al., 2010). In humans, finasteride and dutasteride are reported to have a higher affinity for the 5α-reductase subtype 3 than subtypes 1 and 2 with dutasteride having the lowest IC_50_ (Azzouni et al., 2012). Furthermore, dutasteride has similar affinity for 5α-reductase subtypes 1 and 2, whereas finasteride has more affinity for 5α-reductase subtype 2 (Azzouni et al., 2012). By contrast in rats, finasteride is a potent inhibitor of both types 1 and 2 5α-reductases (Thigpen and Russell, 1992; Azzolina et al., 1997), thus limiting the identification of the relative implication of each isoform in the metabolism of neurosteroids and the assessment of the molecular basis of the neuroprotective effect.

Our previous study in these mice showed that dutasteride but not finasteride protected against the MPTP-induced loss of DA markers (striatal DA and metabolite contents, DA transporter, and vesicular monoamine transporter 2 binding) in the MPTP mouse model (Litim et al., 2015). In the myenteric plexus of these mice, the present results showed that both dutasteride and finasteride protected enteric DA neurons against MPTP toxicity. The fact that the brain contains more 5α-reductase subtypes 1 than 2 while in the small intestine, subtype 2 is more abundant, could explain in part why finasteride (with more affinity than dutasteride for subtype 2 than 1) was more protective in the gut than in the brain. In rodent, finasteride is a potent inhibitor of both 5α-reductase subtype 1 than 2 (Thigpen and Russell, 1992; Azzolina et al., 1997), limiting the identification of the relative contribution of each subtype to the neuroprotective effect in our mice.

MPTP reduced the androgens testosterone and dihydrotestosterone plasma levels of these mice (Litim et al., 2015). This reduction has been already reported not only in the plasma but also in the brain of MPTP mice (Litim et al., 2017a). This result is relevant to the MPTP mouse model of PD and models the human disease where low plasma testosterone levels are reported in men suffering from PD (Okun et al., 2002; Okun et al., 2004a; Okun et al., 2004b). The decrease of testosterone plasma levels in MPTP male mice has been associated with a decrease of Leydig cells’ number as well as ultra-structural alterations in the spared Leydig cells (Ruffoli et al., 2008). In rats, 5α-reductase isoenzymes 1 and 2 are reported to be regulated positively by androgens with decreased mRNA levels in castrated rats (Torres and Ortega, 2003a; Torres and Ortega, 2003b). Thus, 5α-reductase enzymes, the targets of finasteride and dutasteride, are likely decreased in MPTP mice.

VIP is an inhibitory neuropeptide inducing colonic relaxation by secretory effects on the colonic epithelium and is well documented to have anti-inflammatory activities (Chandrasekharan et al., 2013; Morell et al., 2017). In PD patients, impaired colonic motor and rectal sensory functions were reported to be associated with a decrease in VIP expression in submucosal neurons (Giancola et al., 2017), whereas an earlier study showed no change in VIP expression along the length of the gastrointestinal tract (Annerino et al., 2012). In MPTP mice, VIP was shown to protect nigrostriatal DA neurons from death (Delgado and Ganea, 2003; Olson et al., 2015). Moreover, we previously showed a strong colocalization between TH^+^ and VIP^+^ enteric neurons and a decrease in these enteric VIP^+^ neurons in MPTP mice (Côté et al., 2015a; Côté et al., 2015b). In the present study, we show for the first time that both finasteride and dutasteride treatments prevented the loss of VIP neurons in a model of PD.

High levels of proinflammatory markers have been reported many times in PD patients and in the MPTP model (Côté et al., 2015b; Rocha et al., 2015; Pajares et al., 2020; Zhang et al., 2021). We previously observed that estrogenic drugs prevented the inflammatory response in the myenteric plexus of MPTP mice (Côté et al., 2015a; Poirier et al., 2016b). The present study showed an increase of total and proinflammatory enteric macrophages in MPTP mice was prevented by dutasteride, whereas the effect of finasteride treatment was not conclusive. This suggests that the neuroprotective effects of finasteride were not mediated through a regulation of proinflammatory markers. It was hypothesized that the steroid synthetic pathway could be diverted to produce more estrogens by inhibition of the 5α-reductase transformation of testosterone into dihydrotestosterone. Blocking 5α-reductase activity also inhibits progesterone metabolism, a steroid shown previously to be neuroprotective in the MPTP mouse brain (Bourque et al., 2016; Litim et al., 2017b). However, the mechanisms of dutasteride for the neuroprotection could not be related to the modulation of steroids in the plasma and the brain of MPTP mice (Litim et al., 2017b). Hence, other activities of dutasteride or other steroids are likely implicated in its anti-inflammatory activity (di Michele et al., 2013).

Defective mitochondrial activity was reported in PD, including an impairment of the mitochondrial respiratory chain (Grünewald et al., 2019). Accumulating evidence is linking proteins encoded by PD-associated genes to disturbances in mitochondrial function (Gladkova et al., 2018; Borsche et al., 2020). The present study investigated the mitochondrial activity in THP1-XBlue cells which was impaired by MPP^+^ to model the mitochondrial respiratory dysfunctions in PD. Interestingly, we showed that the MPP^+^-induced mitochondrial impairment was prevented by dutasteride but not finasteride treatment in monocytic cells and not in human neuroblastoma SH-SY5Y cells.

The calcium-dependent formation of the mitochondrial permeability transition pore is considered to initiate the apoptotic pathway of various neurodegenerative conditions (Kalani et al., 2018; Rottenberg and Hoek, 2021). Moreover, previously Soskić et al. (2008) showed that ANT-1/2, a component of the mitochondrial permeability transition pore, was the major protein interacting with their dutasteride-biotinylated affinity tag. Hence, in this study, we propose that a molecular interaction of dutasteride with ANT-1/2 could inhibit the mitochondrial permeability transition pore, therefore protecting macrophages against toxic conditions. Of interest, dutasteride did not prevent the alteration of mitochondrial respiratory chain in SH-SY5Y cells, suggesting that the neuroprotective effect of dutasteride is not related to an interaction with mitochondria in these cells. This is in agreement with Isenbrandt et al.’s (2021) study showing that neuroprotective effect of dutasteride in vivo requires the presence of gonadal hormones.

In conclusion, this study showed that dutasteride treatment prevented enteric neuronal damages in the MPTP mouse model with a better efficiency than finasteride. A shorter half-life of finasteride compared to dutasteride could have contributed to its lower neuroprotective activity. We observed in this study that the 5α-reductase inhibitor could have anti-inflammatory effects by targeting the mitochondria of immune cells. This suggests that drug repurposing of dutasteride might be a promising prophylactic avenue in familial forms of PD to treat enteric neuroinflammation to delay disease progression. For future studies, finasteride and dutasteride should be also tested in synucleinopathy models of PD to better assess the therapeutic potential of these drugs in the etiopathogenesis of PD.

## Data Availability

The raw data supporting the conclusions of this article will be made available by the authors, without undue reservation.
