# Supplementary material for: Peripheral Neuroprotective and Immunomodulatory Effects of 5α-Reductase Inhibitors in Parkinson’s Disease Models
Source: Front Pharmacol. 2022 Jul 22;13:898067. doi: 10.3389/fphar.2022.898067 (PMC9355275; doi:10.3389/fphar.2022.898067)
Supplement: Supplementary file 1 [file DataSheet1.docx]

Supplementary Material


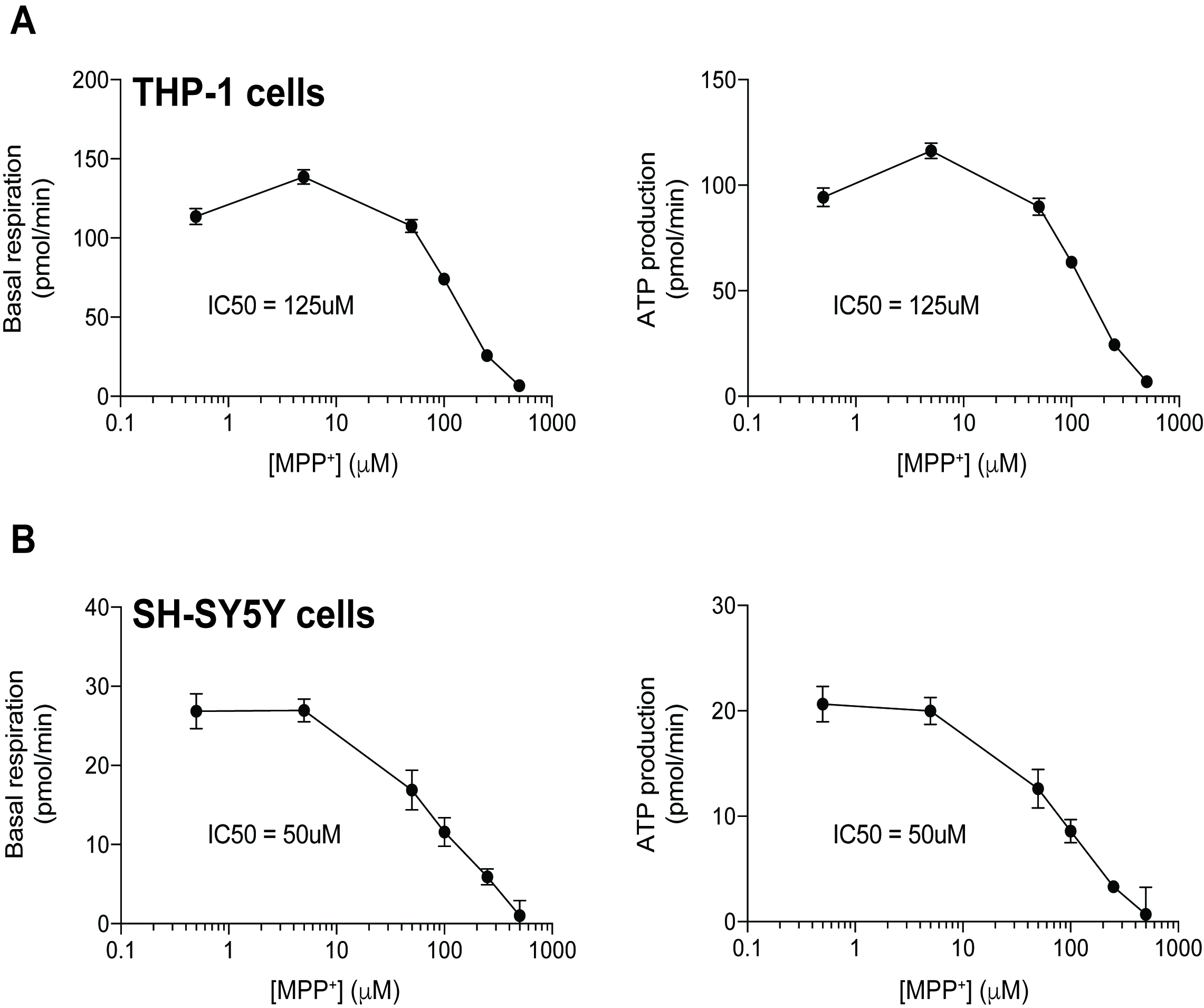


**Figure S1. Dose-response of MPP^+^ on oxygen consumption rate in THP-1 and SH-SY5Y cells.**

Dose-responses of MPP^+^ on mitochondrial basal respiration and ATP production in Seahorse assays. The graphs show the half maximal inhibitory concentration (IC_50_) of both parameters by MPP^+^ in **A)** THP-1 and **B)** SH-SY5Y cells. The concentrations evaluated were 0.5 µM, 5 µM, 50 µM, 100 µM, 250 µM, and 500 µM. The IC_50_ doses chosen were 125 µM MPP^+^ in THP-1 cells and 50 µM MPP^+^ in SH-SY5Y cells.


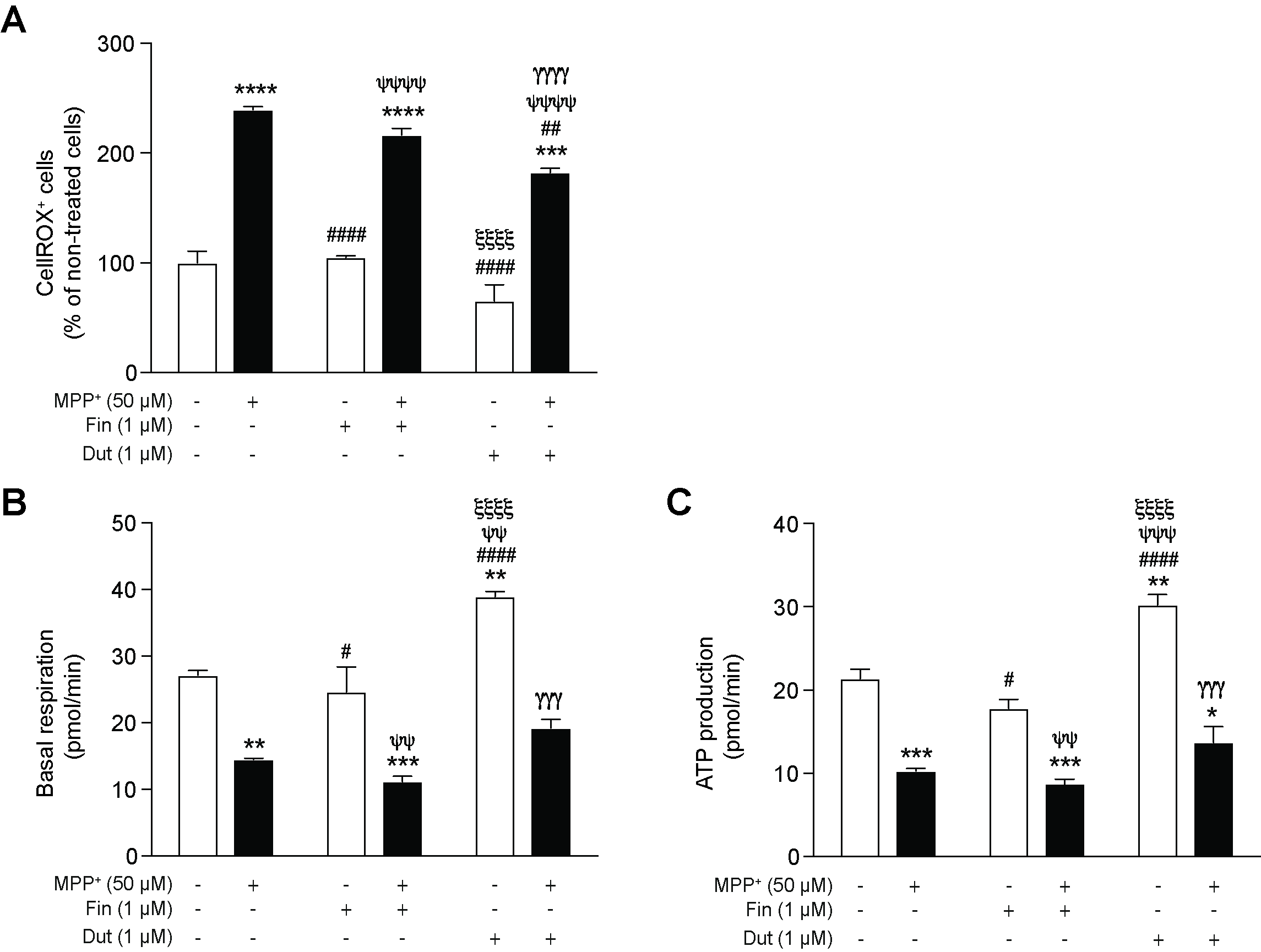


**Figure S2. Cellular oxidative stress and mitochondrial oxygen consumption rates in SH-SY5Y cells.**

SH-SY5Y neuroblastoma cells were treated with finasteride (Fin) or dutasteride (Dut) in the presence or the absence of MPP^+^. Histograms show **A)** the number of positive SH-SY5Y cells to the oxidative stress marker CellROX Orange as percentage of cells and **B)** mitochondrial basal respiration and ATP production quantification in pmol/min. Results are the mean of three experiments. ^*^ *p* < 0.05, ^**^ *p* < 0.01, ^***^ *p* < 0.001 and ^****^ *p* < 0.0001 compared to control; ^#^ *p* < 0.05, ^##^ *p* < 0.01 and ^####^ *p* < 0.0001 compared to MPP^+^ treatment; ^ψψ^ *p* < 0.01, ^ψψψ^ *p* < 0.001 and ^ψψψψ^ *p* < 0.0001 compared to finasteride only; ^ξξξξ^ *p* < 0.0001 compared to MPP^+^+ finasteride. ^γγγ^ *p* < 0.001 and ^γγγγ^ *p* < 0.0001 compared to dutasteride only.
